# Midterm outcomes of the covered endovascular reconstruction of the aortic bifurcation for aortoiliac occlusive disease in a latinoamerican population

**DOI:** 10.1016/j.ijscr.2021.106572

**Published:** 2021-11-03

**Authors:** Luis Fernando García, Juan Carlos Gómez-Rodríguez, Luis Felipe Cabrera-Vargas, Martin Contreras, Ivan David Lozada-Martínez, Sabrina Rahman

**Affiliations:** a Department of Vascular and Endovascular Surgery, Hospital Militar Central, Universidad Militar Nueva Granada, Bogotá, Colombia; b Department of Surgery, Hospital San Rafael, Universidad Militar Nueva Granada, Bogotá, Colombia; c Medical and Surgical Research Center, School of Medicine, University of Cartagena, Cra. 50 #24120, Cartagena, Colombia; d Department of Public Health, Independent University-Bangladesh, Dhaka, Bangladesh

**Keywords:** Covered Endovascular Reconstruction of Aortic Bifurcation, Aortoiliac occlusive disease, Endovascular reconstruction, Aortic bifurcation

## Abstract

**Introduction:**

Surgical approach of aortoiliac occlusive disease (AOD) with aorto-bi-femoral graft or endarterectomy, has been the first line treatment with patency rates up to 90%. Nevertheless, this procedure has an early mortality rate of 4%. Vascular complications of aorto-bi-femoral graft have an average incidence of 5–10% and development of incisional hernia in 10% of the cases. The Covered Endovascular Reconstruction of Aortic Bifurcation or CERAB technique, as a new approach is shaping up to be a promising approach. However, there are few studies in Latin America and the Caribbean.

**Materials and methods:**

Retrospective multicenter study. All patients treated with the CERAB technique between February 2015 and June 2021 in three hospitals.

**Results:**

A total of 9 patients (5 male and 4 female) were treated with the CERAB technique. Only one patient died. Of the total number of patients, 41.2% had a TASC II - C classification, and 58.8% had a TASC II - D classification. Complications included dissection in only 2 patients, massive bleeding in 1 patient and hematoma in 3 patients. The average number of days in critical care was 1.2 days and 2.6 in hospitalization. Two patients required endovascular reintervention. Primary patency was present in 66.7% of the patients.

**Discussion:**

The CERAB technique presents a low morbidity and mortality with an 88.9% of technical success rate. None of our patients needed Chimney CERAB procedure. Our results are similar to those reported in the literature, where they report primary patency rates between 82% and 97%.

## Highlights

• CERAB technique for TASC II C and D patients is safe and feasible.
• CERAB technique is an alternative for open surgery in Latin America.
• CERAB technique has primary patency rates between 82% and 97%.

## 1 Introduction

The aortoiliac occlusive disease (AOD) could lead an entire spectrum of symptoms as the Leriche syndrome that presents with a triad of claudication, impotence, and absence of femoral pulses [1], [2], [3]. Surgical approach with aorto-bi-femoral graft or endarterectomy, has been the first line treatment for AOD with patency rates up to 90%. Nevertheless, this procedure has an early mortality rate of 4%. Vascular complications of aorto-bi-femoral graft have an average incidence of 5–10% and development of incisional hernia in 10% of the cases [4], [5], [6], [7]. Therefore, Goverde et al. [8] identified the need to perform a minimally invasive treatment approach for AOD with high patency rates in the long term and lower morbidity and mortality compared to open approach. Goverde et al. [8] developed in 2009 the Covered Endovascular Reconstruction of Aortic Bifurcation or CERAB technique, as a new approach for extensive and/or recurrent AOD using three covered balloon expandable stents to reconstruct the aortic bifurcation. He published the first experience with CERAB in 2013, showing that this technique provides the ability to deal with TransAtlantic Inter-Society Consensus (TASC II) C and D lesions, simulating a neo-bifurcation or flow divider in combination with the benefits of covered stents [8], [9].

The kissing stent (KS) technique, using two stents abutting or “kissing” in the central lumen of the distal aorta, is the most commonly used endovascular approach for AOD [9]. The main issue with this endovascular approach is its geometrical configuration that was identified as a risk factor for restenosis and thrombosis with primary patency at 2-year follow-up of 79% and 48% in TASC II C and D lesions respectively [1], [2], [3]. The cross-configuration in KS influences the mismatch areas between the stent and vessel wall that in turn cause flow disturbances [1]. This mechanical phenomenon develops neointimal hyperplasia induced by low oscillating wall shear stress and stagnant blood flow [2], [3]. The recent evidence shows a technical success rate for CERAB above 90% with a primary patency at one year of 87% for TASC-C and 90% for TASC-D lesions, respectively with a shorter hospital stay compared to aorto-bi-femoral graft or endarterectomy [9], [10], [11], [12]. The aim of this study was to evaluate the 2-year outcomes of the first Colombian and Latinoamerican series of CERAB. This case report followed the PROCESS guidelines for its realization [13].

## 2 Materials and methods

This study was approved by the ethics committee of the Universidad Militar Nueva Granada. We conducted a retrospective multicenter case series. All patients treated with the CERAB technique between February 2015 and June 2021 in three hospitals, Hospital Militar Central, Clinica Marly and Clinica Colombia, were identified and analyzed retrospectively. Human investigation review board approval was obtained for this study, and informed consent of the patient was not required because of the retrospective design. Patients with symptomatic AOD TASC II A, B, C or D, non-active smokers at least for 3–6 months, unfit for open surgery, availability to continue with imaging long term follow up, best medical treatment adherence, and multiple comorbidities were selected to the CERAB procedure. Patients treated for acute limb ischemia and patients with chimney configurations were excluded from the analysis. Before endovascular treatment, all patients were treated with antiplatelet therapy, oral anticoagulation therapy, statins, and supervised walking exercise. Medical files were screened for demographic data, clinical status (using the Fontaine classification for chronic ischemia) [14], complications, and information on follow-up. Lesions were categorized according to the TASC II criteria [15] by assessing the computed tomography angiography (CTA) scans. Procedural reports were used to extract information on the procedure and the stent types used. Follow-up was scheduled after 6 weeks and 6, 12 and 24 months and consisted of clinical assessment and duplex ultrasound with ankle-brachial index (ABI) measurements.

### 2.1 CERAB technique

**Fig. 1:**
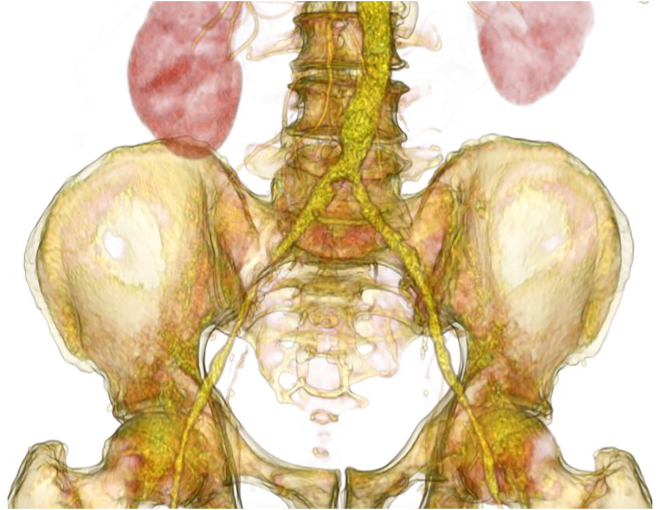
3D images of TASC II C aorto iliac occlusive disease.

**Fig. 2:**
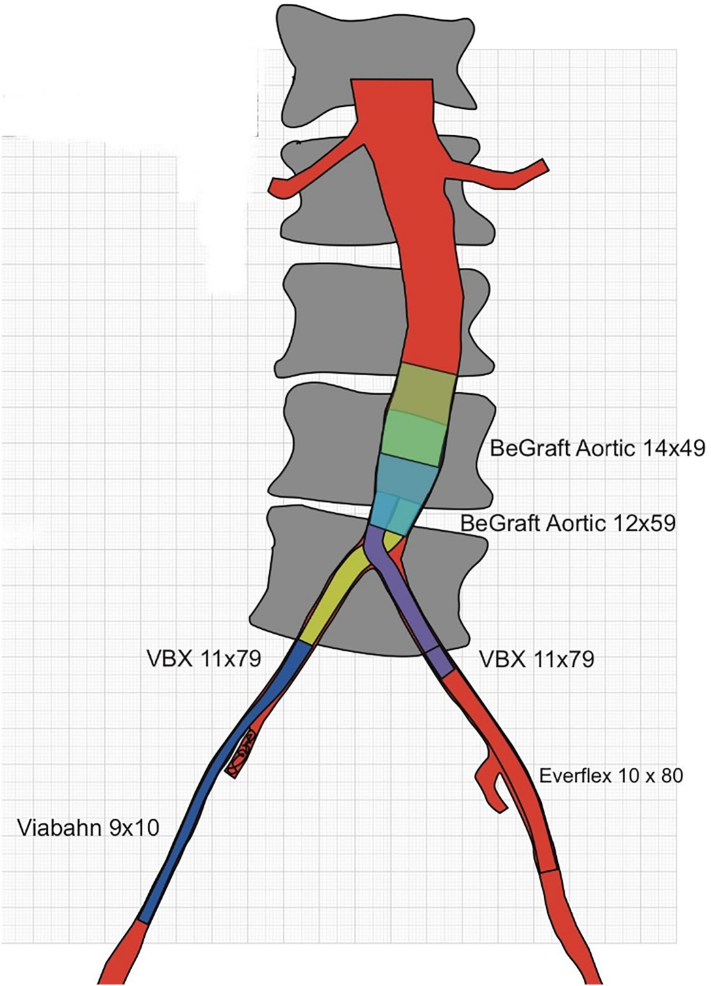
CERAB construction: different stents graft uses to cover the entire aorto iliac bifurcation.

**Fig. 3:**
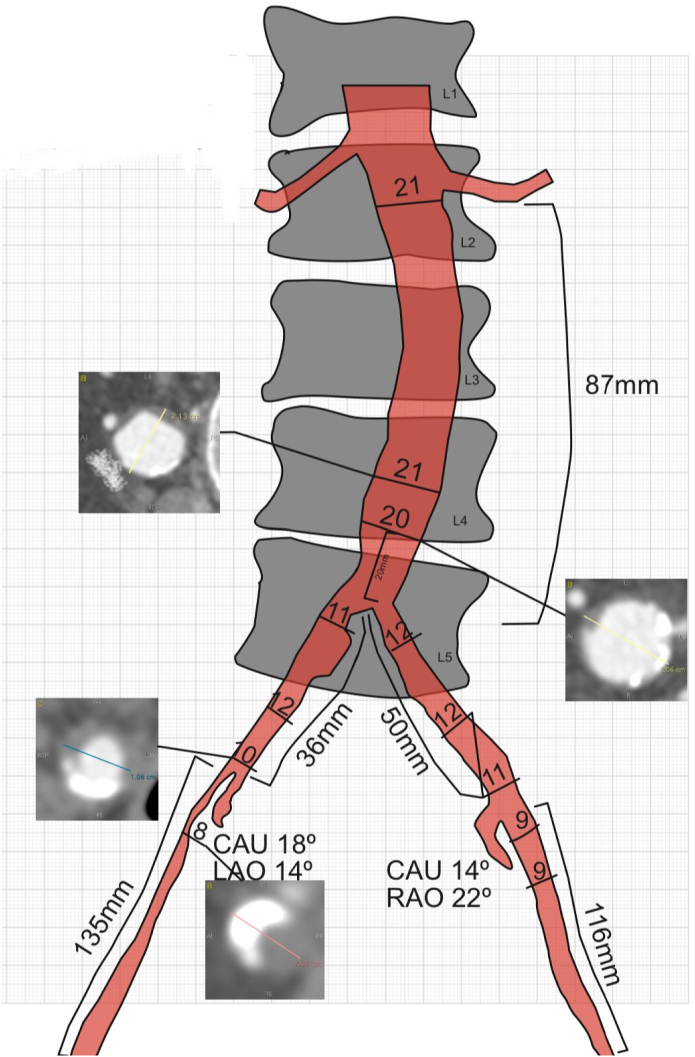
Preoperative image endovascular surgical planning for CERAB.

Suitability for the technique was evaluated on the basis of CTA imaging (Fig. 1, Fig. 2, Fig. 3). Two introducer sheaths are placed in the common femoral arteries. The lesion is crossed either endoluminally or subintimally, depending on the lesion's characteristics. After predilation, a 9F introducer sheath is inserted above the proximal margin of the aortic lesion. Thereafter, a 12-mm balloon expandable expanded polytetrafluoroethylene covered stent (Bentley BeGraft aortic) is deployed in the distal aorta. The distal end of the stent is placed approximately 15 to 20 mm above the bifurcation to facilitate canalization. This stent is flared proximally with a balloon adapted to the native diameter of the distal aorta, typically with a diameter of 16 to 20 mm, to ensure full apposition to the aortic wall. This creates a funnel-shaped stent with a distal segment that is still 12 mm in diameter. Subsequently, two iliac covered stents, typically 8 mm, are positioned in the conic segment and simultaneously inflated using the KS technique (Gore Viabahn VBX). As treatment planning is always from healthy-to-healthy tissue, in some cases distal extensions are required. In these cases, we try to preserve the internal iliac arteries, if patent, using a bare-metal stent (Bard LifeStream) at these locations to prevent buttock claudication and erectile dysfunction. After the procedure, patients receive oral anticoagulacion, statin treatment and dual antiplatelet therapy for at least 6 months, after which single antiplatelet and statin therapy is continued.

### 2.2 Outcomes

Primary outcome of the study was the 2-year primary patency. Secondary outcome measures included assisted primary patency, secondary patency, technical success, clinical improvement, length of hospital stay, 30-day morbidity, mortality, and secondary interventions. Patency was determined by means of duplex ultrasound (peak systolic velocity [PSV] <2.5). Primary patency was defined as patency that is obtained without the need for additional or secondary surgical or endovascular procedures [1]. Assisted primary patency is defined as patency of the configuration achieved with the use of an additional or secondary surgical or endovascular procedure, as long as occlusion of the treated segment has not occurred [1]. Secondary patency was defined as the patency achieved by all procedures to recanalize an occluded CERAB configuration, preserving the configuration. Technical success was achieved when blood flow was restored with <30% residual stenosis [1]. Restenosis was defined as a PSV ratio >2.5, measured proximal to, in, or distal to the graft on duplex ultrasound.1 Major and minor complications were registered up until 30 days after the procedure.

### 2.3 Data analysis

Continuous variables were reported as medians with interquartile ranges (IQR). Categorical variables were reported as numbers and percentages. All analyses were performed using Excel Office (Microsoft, Washington, DC).

## 3 Results

During the study period, a total of 9 patients (5 male and 4 female) were treated with the CERAB technique at the three institutions. Characteristics of the patients are depicted in Table 1, perioperative outcomes and patency rates are shown in Table 2, Table 3. The maximum follow up was 6 years for the first patient. The CERAB technique presents 88.9% of technical success rate with a 2 years primary patency rate of 66.7%. The main indication for the CERAB technique in the 66% of the patients in this study was intermittent claudication, not responding to conservative treatment. There were 2 early occlusions of the CERAB that were successfully treated by thrombectomy or thrombolysis. One patient presented the CERAB occlusion after 16 days and the other one 21 days of the procedure. The main complications reported in our study were dissection in 22.2% and groin hematoma in 33.3% of the patients without need of re-operation. One of our patients developed an acute kidney injury.

**Table 1:** Baseline patient characteristics.

Variables (%)	Total (*n* = 9)
Men	52.9
Age (years)	66.3 (RIQ 3.2)
Comorbidities	
Diabetes mellitus	94.1
Smoking	100
Pulmonary disease	52.9
Cardiac disease	76.4
Carotid disease	17.6
Renal disease	11.7
ASA category	
1	0
2	17.6
3	82.4
4	0
Fontaine classification	
I	0
IIA	0
IIB	70.6
III	29.4
IV	0
TASC II classification	
A	0
B	0
C	41.2
D	58.8

**Table 2:** Perioperative outcomes.

Variables (%)	Total (n = 9)
Surgical endovascular technique (n)	
Polytetrafluoroethylene covered stents	6
Aortic endograft	3
Surgical time (mins)	193 (RIQ 35)
Bleeding (cc)	85 (RIQ 11)
Intensive care unit (days)	1.2 (RIQ 0.3)
Hospital stay (days)	2.6 (RIQ 1.2)
Acute renal insufficiency	11
Complications	
Dissection	22.2
Massive bleeding	11.1
Stent deformation	0
Dislocation of stent	0
Thrombus formation	22.2
Wound infection	0
Groin hematoma	33.3
Conversion to open surgery	11.1
Reintervention	
Endovascular	22.2
Open	0
Mortality	11.1

**Table 3:** Two years patency results.

Variables (%)	Total (n = 9)
Primary patency	66.7
Primary assisted patency	0
Secondary patency	33.3

## 4 Discussion

In this study, we have demonstrated that the CERAB technique for TASC II C and D patients is safe and feasible with a 2 years primary patency rate of 66.7% in our latinoamerican population, avoiding the open surgical approach and its higher rate of perioperative complications. The CERAB technique presents a low morbidity and mortality with an 88.9% of technical success rate. None of our patients needed Chimney CERAB procedure. Our results are similar to Taeymans et al. [1] and Dorigo et al. [16] with primary patency rates between 82% and 97% [1], [16].

The most recent publication about the CERAB technique for AOD is the study of Taeymans et al. [1] in 2018. Nevertheless, the clinical evidence on CERAB is still limited to case series. Taeymans et al. [1] described a series of 130 elective CERAB surgeries, excluding Chimney procedures [1]. The 53% of patients in this study were male with an average age of 61 years old, similar to our population. None of our patients had previous aorto iliac interventions but in this cohort, 41% of the patients had a previous surgery, including open surgery in 7 and an endovascular treatment in 46 patients. The main indication for the CERAB technique in the 66% of the patients in this study was intermittent claudication, not responding to conservative treatment and the remaining patients were treated for critical limb ischemia [1]. The 89% of the patients had a TASC II D classification, similar to our results. The ankle–brachial index significantly increased from 0.65 ± 0.22 to 0.88 ± 0.15 with a 30 days major complications rate of 8% and no 30-day mortality, similar to our study. The principal reported complication was a stent collapse in one of the limbs in three patients of which two were salvaged endovascular [1]. In our series there was no report of stent collapse but these early collapses could indicate that the stent design is still not optimal to overcome the often heavily calcified lesions and the ideal stent for the CERAB technique is still not developed.

The key point to avoid stent collapse is a proper completion imaging with an angiography in two directions or a cone beam computed tomography to prevent a suboptimal stent deployment during the procedure. Only one patient had an acute renal insufficiency without dialysis requirement. There were two early occlusions of the CERAB that were successfully treated by thrombectomy or thrombolysis. One patient presented the CERAB occlusion after 16 days and the other one 21 days of the procedure, reconsulting to the emergency department and underwent endovascular revision. We presented one death with massive bleeding due to a right common iliac artery rupture that was not possible to control with an open conversion. The main complications reported in our cohort were dissection in 22.2% and groin hematoma in 33.3% of the patients without need of re-operation. One of our patients developed an acute kidney injury. On the other hand, the aorto-bi-femoral graft has a vascular complications rate of 5–10%, development of incisional hernia in 10% of the cases and mortality rate of 4%. This phenomenon may be explained due to the low number of cases of CERAB procedures that limit the surgeons to reach que learning curve. We presented a similar median hospital stay of two days. In the Taeymans et al. [1] series, 96% of the patients clinically improved at least one Rutherford category with a limb salvage rate of 97% at three years of follow-up [1], [17], [18], [19].

In vitro data have shown that the CERAB configuration is geometrically superior to kissing stent endovascular approach with a more favorable flow conditions [20], [21]. The kissing stents technique could be used effectively as long as they do not protrude too far in the distal aorta [3], [20], [21]. There are no prospective comparative trials of kissing stents vs CERAB technique. In the meta-analysis of Groot et al. [17] on the treatment of kissing stents, they reported less complex patients with lower TASC II classification and less previous interventions than the recent publicated series of CERAB, as in our study where all the patients were TASC II C and D. If we compare this data with ours and the Taeymans et al. [1] series, the primary and secondary patency rates were better in the CERAB cohort. As an alternative for balloon-expandable stents, the use of self-expanding stents in a y-configuration has been suggested but in our series all cases of CERAB were performed using balloon-expandable stents cannulating the aortic stent through the femoral access. Nevertheless, in a young and fit patient, the surgical option may be better, as the long-term outcome of CERAB technique is unknown [1], [2], [18].

Alternative techniques for the CERAB approach in AOD are reported in the literature, as the use of aortic endograft developed to treat abdominal aortic aneurysms, published in 2005 by Maynar et al. [19], a series of five patients with TASC-C or TASCD iliac occlusions successfully treated with the Gore Excluder Endoprosthesis (W.L. Gore and Associates) [19] just like the first 3 cases of our study where we used an aortic endograft to treat the AOD. In 2016, Maldonado et al. [18] reported a series of 91 patients treated with the AFX Unibody Endograft (Endologix, Irvine, CA, USA). The outcomes were comparable with our CERAB series with regard to clinical state and TASC II classification. Nevertheless, these aortic endografts have a larger profile and should not be pulled onto an occluded or stenosed bifurcation. Moreover in low- and middle-income countries like ours, the balloon-expandable covered stents allow the surgeon to reach large diameter aortas with a lower cost and a higher availability. The CERAB technique due to its minimally invasive context and its short ICU and hospital stay could be a cost effective approach for latinoamerican countries like ours and specially during this new COVID-19 era were the hospitals needs more free ICU beds [2], [18], [19].

A possible disadvantage of the use of covered stents is the overstenting of collateral arteries, as this might generate occlusion. The preservation of a permeable hypogastric artery could prevent ischemic complications such as buttock claudication. In our cohort we tried to preserve the patent iliac internal artery using a bare-metal stent (Bard LifeStream) [22]. Until now the data about the CERAB technique consist of retrospective series of a small group of experienced hospitals. Large multicentric prospective studies on the CERAB approach are needed.

The findings of this study should be interpreted within the context of its design. It's a retrospective multicenter non randomized study with a small sample and no control group. The results should therefore be viewed as hypothesis-generating to conduct future studies. All data were retrospectively collected from the electronic medical records and the outcomes are based on what has been registered. This study presents the risk of selection bias and the limitations secondary to the lack of individual clinical patient information, prone to recall bias or misclassification bias. Strengths of this study are the detailed short and long-term clinical outcomes of CERAB technique in a latinoamerican population and the patients follow up.

## 5 Conclusions

The CERAB technique is a low to moderate morbidity endovascular approach related to good 2-year outcomes in a latinoamerican population with a more anatomical and physiological method to reconstruct the aortic bifurcation. Nevertheless, long-term large multicentric prospective comparative studies are indicated to evaluate its superiority over kissing stents and open surgical approach.

## Abbreviations

ABI: ankle-brachial index
AOD: aortoiliac occlusive disease
CERAB: Covered Endovascular Reconstruction of Aortic Bifurcation
COVID-19: coronavirus disease 2019
CTA: computed tomography angiography
KS: kissing stent
PSV: peak systolic velocity
TASC II: TransAtlantic Inter-Society Consensus

## Declaration of competing interest

The authors declare that they have no known competing financial interests or personal relationships that could have appeared to influence the work reported in this paper.
